# Myositis “Diaphragm Cramp” as a Potential Cause of Respiratory Arrests in Infants. Comment on Salfi, N.C.M. et al. Fatal Deterioration of a Respiratory Syncytial Virus Infection in an Infant with Abnormal Muscularization of Intra-Acinar Pulmonary Arteries: Autopsy and Histological Findings. *Diagnostics* 2024, *14*, 601

**DOI:** 10.3390/diagnostics14101061

**Published:** 2024-05-20

**Authors:** Dov Jordan Gebien

**Affiliations:** Rejoo Medical Clinic, 3319 Bayview Ave, Toronto, ON M2K 1G4, Canada; survivingsids@gmail.com

This Letter to the Editor provides additional information regarding the tragic case of a 6-month-old in Italy with respiratory syncytial virus who deteriorated and died unexpectedly from rapid respiratory insufficiency [1]. It highlights the differences between fatigue of the primary inspiratory muscle, the diaphragm, and outright pump failure. It also discusses peculiar, unexplained histopathological findings and therefore highlights the need to include diaphragm histology in all autopsies in sudden unexpected deaths, especially with coexistent infections.

In their informative write up, the authors alluded to the importance of close observation for apnea in infants with bronchiolitis, particularly preterms and those under 3 months given they are at risk for rapid respiratory distress and what is effectively sudden unexplained infant death. Javouhey et al. (2013) noted that the most common clinical presentations of severe bronchiolitis are acute hypercapnic (Type II) respiratory distress (resulting from inspiratory muscle fatigue and alveolar hypoventilation) and recurrent, severe apnea [2]. Respiratory fatigue occurs in both; however, the latter could also be caused by a lack of CNS stimulation to the respiratory muscles (central apnea). Apnea from fatigue-induced “critical diaphragmatic failure” (peripheral apnea) was proposed to be causal in sudden infant death syndrome (SIDS) in 2011 [3]; however, for inexplicable reasons, it has not been investigated despite its merit. Many characteristics associated with apnea in RSV overlap with factors that contribute to diaphragm fatigue. These are listed in Table 1 and include younger infants (underdeveloped respiratory muscles, pliable chest wall, less fatigue-resistant myofibers), hypoxemia, hypercapnia and acidosis (both respiratory and metabolic types) amongst other factors like prone positioning, nicotine exposure and REM sleep (not listed) [3]. Importantly, these are all SIDS risk factors as well, which can be broadly classified under prematurity and young infancy, rebreathing exhaled gases, nonlethal infections, tobacco smoke exposure and more. This is not the first paper to describe this overlap.

Although most children with severe bronchiolitis are infected by respiratory syncytial virus (RSV), many other viruses are implicated including rhinovirus, adenovirus, coronavirus and influenza [4]. Histological studies in influenza B-positive children with incapacitating calf pain demonstrated limb (skeletal) muscle myopathic abnormalities marked by highly elevated creatine kinase levels (indicative of disrupted cell membranes, primarily by hypoxia) [5]. Biopsies revealed patchy areas of necrotic, ruptured myofibers with unexpectedly scant inflammation. In comparison in SIDS, the diaphragm (also a skeletal muscle) similarly exhibited focal areas of rupture and necrosis with a lack of acute infiltrates in 82% of cases [6]. Contraction band necrosis was found, its presence indicating some sort of terminal injury had occurred under anoxic conditions, and caused diaphragm sarcomere hypercontraction. Eisenhut (2011) wrote of a case nearly identical to the one referenced here: a 5-month-old admitted with a viral upper respiratory infection and poor feeding who suddenly died in hospital by unexpected respiratory arrest [7]. Although the autopsy showed no gross abnormality, diaphragm histology revealed focal infiltrates and myofiber destruction along with myocyte necrosis and regeneration (no mention of contraction bands). Myocardium, brain and limb muscles were normal. Dr. Eisenhut speculated that diaphragm inflammatory mediators from the viral myositis/myopathy may have disrupted excitation–contraction coupling, leading to the respiratory arrest by diaphragm pump failure.

Although the injury mechanism responsible for contraction band necrosis has never been elucidated, a clue might come from an obscure condition known as diaphragmatic flutter. Also known as van Leeuwenhoek’s disease, it involves episodic, involuntary diaphragm contractions that typically cause tachypnea and dyspnea with uncomfortable abdominal pulsations in adults [8]. In young children, one case report revealed it was well tolerated in RSV-positive infants [9], whereas in others, airway support was temporarily needed in neonates just after birth [10,11]. A literature review revealed that flutter appears to be just one condition along a frequency spectrum of hereby termed “diaphragm hyperexcitability disorders” (DHDs; essentially diaphragm arrhythmias) (Figure 1). Diaphragm fatigue could predispose to these. Respiratory (and psychological) distress becomes prevalent among the higher-frequency conditions. Involuntary belching, retching, hiccups and diaphragm spasms occupy the milder end, whereas myoclonus, low- and high-frequency diaphragm flutter and diaphragm tetany are at the severe end. Examples of the latter include sudden respiratory arrests (diaphragm paralysis apnea) by some electrocutions [12], neurotoxins like ingested nicotine [13] and neuromuscular blockers such as succinylcholine [14]. Unfortunately, diaphragm histology, to determine contraction band necrosis, was unavailable in these deaths (however, in electrocution victims, cardiac contraction bands with myofiber ruptures and contractures were detected) [15].

Although apnea was not found to be a symptom of diaphragm flutter in the literature review, it is thought to occur in a more severe form of DHD. In a child with recurrent nocturnal inspiratory arrest (apneic) episodes with gasping and “excruciating, cramp-like bearhug pain”, spontaneous diaphragm cramping could have been responsible [16]. Although unheard of, the diagnosis is plausible given cramp-induced, contracture-like diaphragm paralysis could cause respiratory arrest. An adult by the time of his report, he reportedly autoresuscitated each time, overcoming the painful bearhug apnea with counterintuitive rescue breaths (essentially breathing out to breathe in using high-pressure, pursed-lip, staccato inspirations). Termed putative “diaphragm cramp-like contracture”, it could be the source of CBN hypercontraction seen in SIDS and, quite possibly, the sudden respiratory arrest of the case patient in Italy.

Whether diaphragm cramp exists or not, DHDs probably leave some evidence in vivo. Like calf myopathy in pediatric influenza, creatine kinase levels could screen for potential respiratory muscle involvement in infants with bronchiolitis (as well as those with apneas, cyanosis and brief, resolved unexplained events). If positive, hospital admission would be warranted with continuous monitoring by respiratory inductive plethysmography [10]. DHDs could be confirmed by diaphragm surface electromyography and/or visualized with bedside ultrasound or video fluoroscopy. Ideally, all such measures would make it easier to confirm and gauge the progression of disease, if not to predict those at risk for respiratory deterioration. Finally, all autopsies in sudden, unexpected deaths should include diaphragm histology.

## Figures and Tables

**Figure 1:**
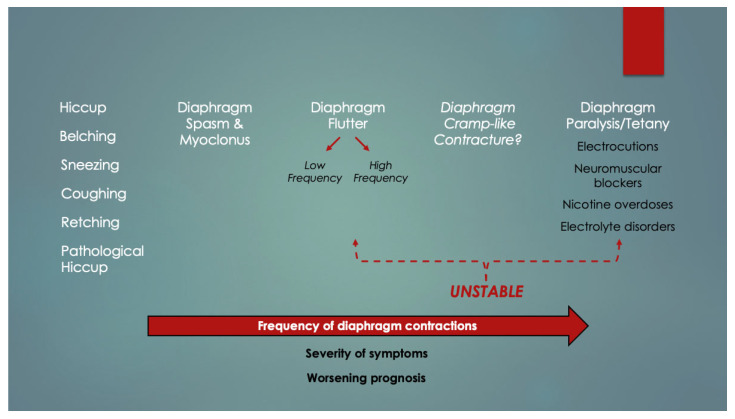
Spectrum of diaphragm hyperexcitability disorders. As the frequency of diaphragm contractions increases, symptoms worsen, and respiratory insufficiency predominates. Flutter can be mildly symptomatic or severe, whereas putative diaphragm cramp-like contracture and tetany exhibit frank respiratory arrests. These could alternatively be termed “unstable diaphragm arrhythmias”.

**Table 1:** Characteristics of RSV-positive infants with and without apnea. The apnea group had apnea at admission whereas the non-apnea group did not. In terms of the diaphragm, low oxygen saturations, hypercapnia and low pH are all thought to induce fatigue with consequent apneas (*peripheral* apneas). If so, a positive feedback cycle would ensue from consequent alveolar hypoventilation (as is evident by the elevated Aa gradient in the apnea group). Pulmonary infiltrates and atelectasis worsen fatigue by increasing work of breathing.

Characteristic	Apnea Group	No Apnea	*p* Value	Author
Younger gestational age at birth (weeks)	33 ± 2	39 ± 2	<0.01	[4]
Younger postconceptional age on admission (weeks)	37 ± 2	53 ± 8	<0.01	[4]
Infiltrates on CXR	5/5 (100%)	8/27 (30%)	<0.02	[4]
Increased alveolar-arterial oxygen gradient (mmHg)	170 ± 96	45 ± 20	<0.01	[4]
Recurrent apneas	18/38 (47%)	6/147 (4%)	<0.005	[5]
Low SaO2 (%)	85 ± 16	90 ± 9	<0.05	[5]
High pCO2 (mmHg)	55.5 ± 18.0	48.8 ± 13.5	<0.05	[5]
Low pH	7.31 ± 0.13	7.36 ± 0.08	<0.05	[5]
Atelectasis on CXR	18/38 (47%)	37/147 (25%)	NA	[5]
Mechanical ventilation	14/38 (37%)	14/147 (10%)	NA	[5]
